# Forward Electric Stimulation-Induced Interference in Intracochlear Electrocochleography of Acoustic Stimulation in the Cochlea of Guinea Pigs

**DOI:** 10.3389/fnins.2022.853275

**Published:** 2022-06-06

**Authors:** Shiyao Min, Tianhao Lu, Min Chen, Jiabao Mao, Xuerui Hu, Shufeng Li

**Affiliations:** ^1^Department of Otolaryngology–Head and Neck Surgery, ENT Institute, Eye & ENT Hospital of Fudan University, Shanghai, China; ^2^NHC Key Laboratory of Hearing Medicine, Fudan University, Shanghai, China

**Keywords:** cochlear implant, interference, intracochlear electrocochleography, electrically evoked compound action potential (ECAP), electric-acoustic stimulation (EAS)

## Abstract

Electric-acoustic stimulation (EAS) uses amplified sound by a hearing aid to stimulate an apical low-frequency region of the cochlea and electrical current from a cochlear implant (CI) to stimulate the basal high-frequency region. EAS recipients had significantly improved speech perception, music appreciation, and hearing function in noise compared to those relying on CI electrical stimulation (ES) alone. However, the interaction between basal ES and apical acoustic stimulation (AS) in the cochlea potentially affects EAS advantages. To investigate ES-AS interaction, we designed a system that recorded the electrically evoked compound action potential (ECAP) and the auditory evoked potential (AEP). We used an intracochlear electrode array to deliver ES at the basal cochlea and detect intracochlear electrocochleography (iECochG) generated from apical AS. Within iECochG, 3 or 6 dB (double or quadruple intensity of ECAP threshold) electric stimulation, 1 ms-forward ES significantly increased CAP amplitudes of 4 kHz/20 dB AS compared to 0 dB ES. Notably, 1 ms-forward 3 dB ES significantly increased CAP amplitudes of 4 kHz/20 dB AS, while 3 or 5 ms-forward ES did not change the CAP amplitudes. The elevation in CAP amplitude of 40 dB/4 kHz AS induced by 1 ms-forward 3 dB ES was significantly lower than that in 20 dB/4 kHz AS. With 1 ms-forward 3 dB ES, AS frequency and stimulating electrode location have no significant impact on relative CAP amplitudes of 20 dB AS. These results suggest that the basal forward ES and the following apical AS could produce a cumulative effect on the auditory nerve response.

## Introduction

Cochlear implants (CIs) have successfully restored hearing perception in numerous adults and children with severe deafness. With the progress of surgery, sound-processing technology, and relaxation of CI applicable standards, numerous patients with low-frequency residual hearing received cochlear implantation. These patients received combined electric-acoustic stimulation (EAS), i.e., electrical stimulation (ES) in the base and middle region of the cochlea and acoustic stimulation (AS) in the apical region of the cochlea. EAS recipients showed considerable improvements in tonal perception (Talbot and Hartley, 2008), speech recognition (Gantz et al., 2005), hearing function against noise background (Incerti et al., 2013), and music appreciation compared to CI recipients using ES alone (Gfeller et al., 2006). However, electrical current delivered by electrodes at the basal or middle region of the cochlea might spread to the apical region and affect the activity of hair cells and nerve fibers there. Consequently, AS in the apical region could be interfered by the ES, which might affect the performance of EAS. Indeed, the interaction between ES and AS was shown to have a negative impact on speech perception (Imsiecke et al., 2020b).

Several previous reports have investigated the psychophysical masking effect between ipsilateral AS and ES in EAS recipients. However, the underlying interaction mechanism between ES and AS is still unclear. Simultaneous ES could produce threshold elevation of AS, which depended on the spatial relationship between ES and AS, i.e., the electric-acoustic frequency difference (EAFD); on the contrary, threshold elevations of ES induced by simultaneous AS were independent of acoustic frequency and electrode location (Lin et al., 2011; Krüger et al., 2017). These studies suggest that the masking effect of simultaneous ES on AS was mostly produced in the cochlea, while acoustic on electric masking may have a more central origin. Therefore, an objective assessment of peripheral EAS interaction would benefit an understanding of its mechanism and characteristics for better EAS performance.

Intracochlear electrocochleography (iECochG) used intracochlear CI electrodes to record acoustically evoked potentials of the hair cells and auditory nerve. The major components of ECochG were the cochlear microphonic (CM) primarily from the outer hair cell stereocilia, the summating potential (SP), and the compound action potential (CAP) from the activity of the auditory nerve fibers. iECochG can be measured by presenting AS with alternating stimulus polarities. The difference between responses to alternating stimulus polarities (CM/DIF) is dominated by the CM and also includes the largest part of the ANN, while the sum of the responses (ANN/SUM) is dominated by the ANN (Forgues et al., 2014; Koka and Litvak, 2017). Recently, several reports used this technology to investigate the electrophysiological characteristics of interference of simultaneous electric stimulation delivery and AS delivery, which were all conducted in human CI recipients (Koka and Litvak, 2017; Imsiecke et al., 2020a; Krüger et al., 2020a,b). These studies suggest that CM/DIF amplitudes to tone bursts could be reduced by simultaneous presentation of an electrical pulse train. Meanwhile, ANN/SUM amplitudes had no significant change, which might be due to that ANN amplitudes were too low to assess a masking effect. A significant decrease of electrically evoked compound action potential (ECAP) with simultaneous AS was also observed (Imsiecke et al., 2020a). Similarly, forward ES with a pulse train could induce behavior threshold elevation of pure tones in an EAFD-dependent manner (Imsiecke et al., 2018). However, electrophysiological characteristics of forward electric stimulation-induced interference in response to AS in EAS recipients were unknown.

Animal studies using normal-hearing or deaf animals with similar cochlear pathological changes could extend our electrophysiological knowledge of electric stimulation interference in responses of AS. Only a few reports studied the electrophysiological characteristics of forward electric stimulation interference in AS (Stronks et al., 2010, 2011, 2013). These studies confirmed that forward electric stimulation with pulse train could suppress afterward pure tone-evoked CAP. However, these studies used an extracochlear electrode at the base of the cochlea or an electrode inserted in the basal turn of the cochlea to deliver electric stimulation. The extracochlear electric stimulation might produce extensive stimulation of the cochlea and its intensity could not be precisely set according to the respective threshold. The single electrode in the base of the cochlea was too far from the apical region to investigate the EAFD-dependent interference from electric stimulation to AS.

To investigate the impact of forward in the basal region of the cochlea on acoustically evoked responses in the apical region of the cochlea, we designed an EAS interaction detecting system capable of delivering combined EAS with adjustable interval and detecting iECochG and ECAP. Using the combined system, we investigated interference from forward ES in the basal region of the guinea pig cochlea to afterward AS in the apical region, as well as the impact of intensity, ES-AS interval, stimulating electrode location, and AS frequency location on it.

## Materials and Methods

### Experimental Animals

Male albino adult guinea pigs with weights ranging from 250 to 300 g were used in the current study. All guinea pigs were confirmed with normal hearing assessed by auditory brainstem responses (ABRs). The following procedures in this study were approved by the Institutional Ethics Committees and ensured adherence to ethical requirements.

### Cochlear Implantation

We employed customized CIs provided by Shanghai Listent Medical Technology Co., Ltd. The implant consisted of four parts, namely, a mini-plug for connecting *in vitro* device, an electrode array with six ring electrodes numbered 1–6 from cusp to tail (E1 to E6), a disk-like electrode (MP2) serving as a reference, and a columnar one (MP1) serving as grounding. Our previous work has described the detailed technical specifications and the following implantation procedure (Li et al., 2020). In brief, after general anesthesia with 16 mg/kg hydrochloride and 16 mg/kg zolazepam, the electrode array was implanted into scala tympani through a round window. MP1 and MP2 were separately placed at an approximately 2 cm distance in the subcutaneous layer of the midline scalp. The approximate distance between the cochlea and each of them was 1.5 cm. The round window membrane was repaired by autologous fascia.

### Combined Electric-Acoustic Stimulation and Intracochlear Electrocochleography Detecting System

**FIGURE 1:**
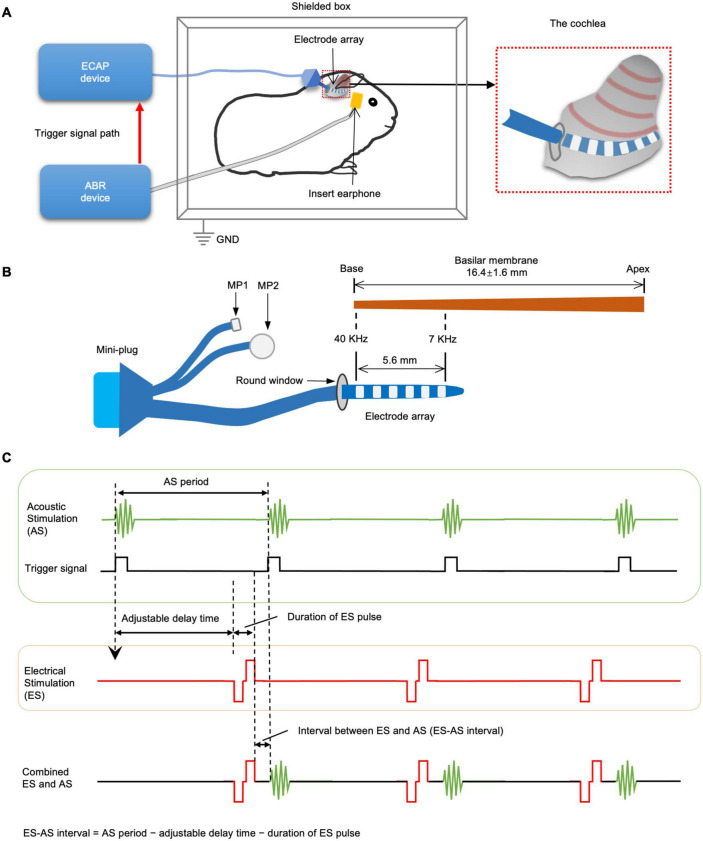
The schematics of combined electric-acoustic stimulation and intracochlear electrocochleography (iECochG) detecting system. **(A)** The guinea pigs were settled in an acoustic and electromagnetic shielding box. ABR recording device delivers acoustic stimulation (AS) through an insert earphone in the external auditory canal. Meanwhile, it delivers trigger signals to a device detecting electrically evoked compound action potential (ECAP) and iECochG. **(B)** The ECAP/iECochG device then delivers electrical stimulation (ES) and records iECochG through electrode array in scala tympani. The intracochlear electrode array has six ring electrodes and connects with the ECAP/iECochG device by a mini-plug and two extracochlear electrodes. The most apical electrode is around 7 kHz frequency site of basilar membrane, while the most basal one is around 40 kHz frequency site. **(C)** The interval between ES and AS (ES-AS interval) can be set by adjusting the delay time between the onset of trigger signal and that of ES. ES-AS interval can be calculated by subtracting the adjustable delay time and ES pulse duration from AS period.

Using an auditory evoked potential (AEP) detecting device and an ECAP detecting device, we established a system delivering combined EAS with the adjustability of stimulation interval and intensity (Figure 1). AS was provided by an ABR device (Natus Medical Incorporated) through insert earphones in auditory canal. ES was provided by sound processor LSP-20B and MAP Version 3.00 software (Shanghai Listent Medical Technology Co., Ltd., Shanghai, China) through one of the intracochlear electrodes and extracochlear MP2. Only the most apical four electrodes were used to deliver ES because they were closer to an exciting region of AS. AS and ES were coupled by a trigger signal from the ABR device to the ECAP device. In this system, the delay of ES onset after receipt of trigger signal could be adjusted to make ES produced at the desired interval before the next AS, which produced combined forward ES and afterward AS. Therefore, ES-AS interval was the AS period subtracted from the adjustable delay time and duration of the ES pulse. AS was set to a repetition rate of 7.7 Hz, which mean interval between two ASs was about 130 ms. ES was a single charge-balanced biphasic pulse with 9 μs interphase gap, 32 μs phase duration, and adjustable intensity. The accuracy of ES-AS interval was calibrated *via* a mixed-signal oscilloscope (MSO-X 4034A, Agilent Technologies, Inc., Santa Clara, CA, United States) with a maximum error of ±0.05 ms.

### Electrically Evoked Compound Action Potential Threshold Measurement

The ECAP detecting device was used to measure both ECAP and iECochG. After implant surgery, the ECAP threshold of each electrode was measured to determine the corresponding intensity of ES which was previously described by Li et al. (2020). This study employed ES with three different intensities of each electrode, i.e., current intensity equal to the corresponding ECAP threshold (ES/0 dB), double ECAP threshold (ES/3 dB), and quadruple ECAP threshold (ES/6 dB).

### Intracochlear Electrocochleography Measurement

**FIGURE 2:**
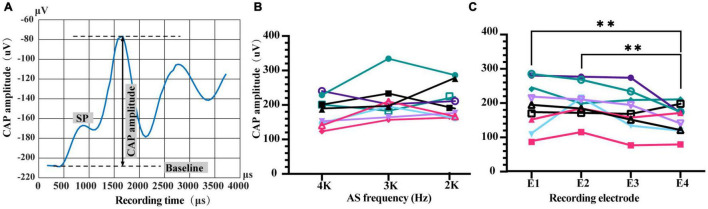
Characteristics of intracochlear electrocochleography (iECochG) using different recording electrodes and AS frequency. **(A)** An example of iECochG recorded. Baseline is a horizontal line starting from the initial point of SP. CAP amplitude is determined as the distance from CAP peak to baseline. SP, summating potential; CAP, compound action potential. **(B)** CAP amplitudes of 20 dB AS with frequencies of 4, 3, or 2 kHz were comparable when recorded by number 1 electrode, *F*(2,16) = 2.553, *P* = 0.1090. **(C)** Recorded CAP amplitudes of 4 kHz/20 dB AS by different electrodes were significantly different, *F*(3,24) = 4.956, *P* = 0.0081. CAP amplitudes recorded by the most basal electrode E4 were significantly lower than those recorded by E1 and E2, *P* = 0.0068 and 0.0016, respectively. Repeated measure one-way ANOVA with Fisher’s LSD multiple comparisons. The lines with different colors in **(B,C)** represent different animals. ** *P* < 0.01.

Combined EAS experiments were conducted in a sound-proof and electrically shielded box. The most apical electrode except stimulating one for ES and extracochlear MP1 electrode was used to record iECochG with a sampling rate of 20 kHz. AS was a tone burst with 1 ms duration, various frequencies (2, 3, or 4 kHz), and intensities (20 or 40 dB above the corresponding iECochG threshold). Data acquired from the MAP version 3.0 software were further processed in MATLAB (version R2012b, MathWorks, Inc., Natick, MA, United States) to generate a waveform of iECochG and measure CAP amplitude. Due to the limitation of sampling duration of hardware, each sampling time window was only 1.6 ms. We repeated the combined EAS three times and measured their responses in three overlapping time windows to extend the sampling duration. They started at 1, 2, and 3 ms after AS onset, respectively, and then stitched to a summed 3.6 ms-duration sampling from 1 to 4.6 ms after AS onset. The number of sweeps for each measurement was 50. CAP amplitude within iECochG was determined as the distance between CAP peak and baseline that was a horizontal line drawn from the SP onset (Figure 2). Intracochlear ECochG threshold was determined as the lowest acoustic intensity with the presence of CAP for each frequency.

### Statistical Analysis

This study aimed to investigate the interference of forward ES in AS responses, mainly referring to changes in the CAP amplitude of iECochG. Even within the same species, CAP amplitude varies between individuals and recording electrodes. In an attempt to reduce the influence of the variance, relative CAP amplitude, i.e., a ratio of CAP amplitude with forward ES to that without interaction, was also used for analysis. Statistical analysis was performed using Prism GraphPad (version 9.3.0). Two-tailed paired Student’s *t*-test and ordinary or repeated measures (RM) one-way analysis of variance (ANOVA) with Tukey’s multiple comparisons test were conducted to evaluate CAP amplitudes’ thresholds in different circumstances. Statistical significance was based on the *p*-value less than 0.05.

## Results

### Feasibility of the Electric-Acoustic Stimulation Interaction Detecting System

The system established in this study successfully provided an availability of EAS with adjustable ES-AS sequence, interval and intensity, and detection of corresponding ECAP and iECochG for the investigation of ES-AS interaction. This study used an intracochlear electrode array (E1–E4) to deliver forward ES at the base of the cochlea, together with an insert phone at the external auditory canal to deliver afterward 4, 3, or 2 kHz AS to excite the more apical region of the cochlea. CAP amplitudes of iECochG induced by afterward AS were measured to assess forward ES interference. First, we investigated the iECochG characteristics of plain AS with different recording electrodes and acoustic frequencies. The CAP amplitudes of AS at 4 kHz frequency with 20 dB intensity above the iECochG threshold (20 dB/4 kHz AS) varied with the change of recording electrode [Figure 2B, *F*(3,24) = 4.956, *P* = 0.0081]. CAP amplitudes recorded by No. 4 electrode (E4) were significantly lower than E1 and E2 (*P* = 0.0068 and 0.0016, respectively). These results suggest that the distance between AS exciting region and the location of recording electrode affected the amount of recorded CAP amplitudes. However, there was no significant difference among CAP amplitudes recorded by E1, E2, and E3 (*P* > 0.05). Meanwhile, CAP amplitudes of 20 dB 4, 3, and 2 kHz AS when using E1 as a recording electrode were also comparable [Figure 2C, *F*(2,16) = 2.553, *P* = 0.1090].

### Impact of Forward Electrical Stimulation Intensity on Its Interference in Intracochlear Electrocochleography of Acoustic Stimulation

**FIGURE 3:**
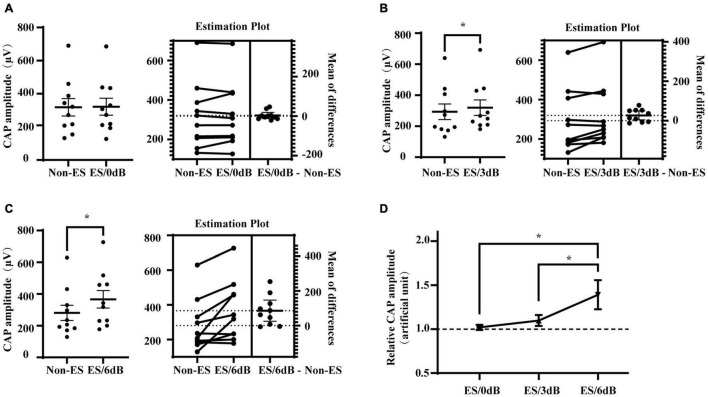
Impact of forward ES intensity on its interference in iECochG of AS. **(A)** One ms forward 0 dB ES (intensity equal to ECAP threshold) from the most apical electrode did not change CAP amplitudes of the following 4 kHz 20 dB AS, *P* = 0.6593. **(B,C)** However, 3 dB (double ECAP threshold intensity) or 6 dB (quadruple ECAP threshold intensity) ES significantly increased CAP amplitudes of following AS, ES/3 dB vs. non-ES, *P* = 0.0271; ES/6 dB vs. non-ES, *P* = 0.0114. In **(A–C)**, each plot means an animal data and the horizontal line means the mean across animals. **(D)** Relative CAP amplitudes varied with ES intensity, *F*(2,18) = 4.342, *P* = 0.0289. Relative CAP amplitudes of AS combined with 6 dB forward ES were significantly higher than that of AS with 3 or 0 dB forward ES, *P* = 0.0284 or 0.0338, respectively. Non-ES, no electrical stimulation; ES/0 dB, ES with an intensity equivalent to a corresponding ECAP threshold level; ES/3 dB, ES with an intensity of 3 dB above the corresponding ECAP threshold, i.e., the double intensity of ECAP threshold level. ES/6 dB, ES with an intensity of 6 dB above the corresponding ECAP threshold, i.e., the quadruple level intensity of ECAP threshold level. Relative CAP amplitudes were ratios of CAP amplitudes with to without forward electrical stimulation. **(A–C)** Treated with two-tail paired *t* test. **(D)** Treated with RM one-way ANOVA followed by one-tail paired *t* test multiple comparisons. *P < 0.05.

To investigate the effect of forward ES with different intensities on iECochG of AS, we compared the CAP amplitudes of 20 dB/4 kHz AS with or without 0, 3, and 6 dB 1 ms-forward ES, respectively (Figure 3). ES/0 dB did not induce a significant change in the CAP amplitudes (*P* = 0.6593). ES with the higher intensities, i.e., ES/3 dB and ES/6 dB, produced significantly higher CAP amplitudes (*P* = 0.0271 and 0.0114, respectively). Relative CAP amplitudes tended to increase when increasing forward ES intensity (ES/6 dB vs. ES/3 dB, *P* = 0.0338; ES/6 dB vs. ES/3 dB, *P* = 0.0284; ES/3 dB vs. ES/0 dB, *P* = 0.0836). These results suggest that combined forward ES and afterward AS could increase detected activities of the auditory nerve. In addition, this effect increased with forward ES intensities.

### Impact of Electrical Stimulation-Acoustic Stimulation Interval on Forward Electrical Stimulation-Induced Interference in Intracochlear Electrocochleography of Acoustic Stimulation

**FIGURE 4:**
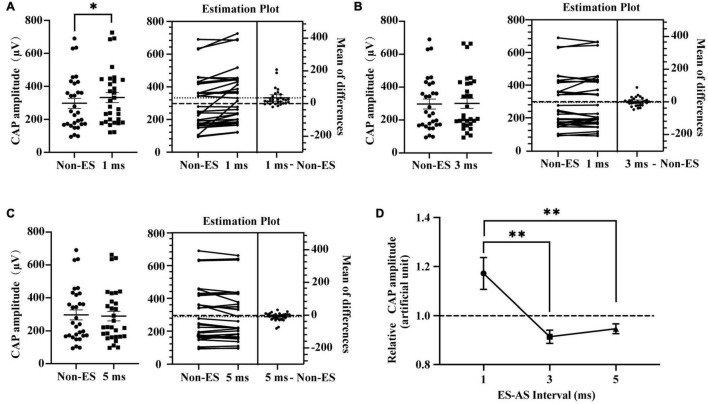
Impact of ES-AS interval on forward ES-induced interference in iECochG of AS. **(A)** One-ms-forward 3 dB (double ECAP threshold intensity) ES with E1 significantly increased CAP amplitudes of 4 kHz/20 dB AS, *P* = 0.0041. **(B,C)** CAP amplitudes in groups with 3 and 5 ms-forward ES were comparable with that in a non-ES group, *P* = 0.4773 and 0.1294, respectively. In **(A–C)** each plot means an animal data and the horizontal line means the mean across animals. **(D)** Relative CAP amplitudes varied with interval between ES and AS, *F*(2,58) = 8.019, *P* = 0.0008. Relative CAP amplitudes of AS combined with 1 ms-forward ES were significantly higher than that with 3 or 5 ms-forward ES, *P* = 0.0015 and 0.0060, respectively, while that in the last two groups were comparable and inclined to be unit, *P* = 0.8887. **(A–C)** Treated with a two-tailed paired *t* test. **(D)** Treated with repeated measures (RM) one-way ANOVA with two-tail paired *t* test multiple comparison. *P < 0.05; ^**^P < 0.01.

To investigate the role of ES-AS interval in ES-induced interference in the cochlear responses to AS, we compared the CAP amplitudes of combined EAS with different ES-AS intervals (Figure 4). The generation of CAP is mainly attributed to the auditory nerve which has been shown to have an absolute refractory period (ARP) of 0.5 ms and a relative refractory period (RRP) of approximately 4 ms (Boulet et al., 2016). Therefore, intervals at 1, 3, and 5 ms were employed in this study (Figure 4). For 20 dB/4 kHz AS, 1 ms-forward 3 dB ES induced a significant elevation of CAP amplitudes when compared to that without forward ES (*P* = 0.0041). However, CAP amplitudes in groups with 3 and 5 ms-forward ES were compared to those in the non-ES group (*P* = 0.4773 and 0.1294, respectively). In addition, relative CAP amplitudes in 1 ms-interval were above unit and significantly higher than those in 3 and 5 ms-interval groups (*P* = 0.0015 and 0.0060, respectively). These results suggest that combined forward ES and afterward AS with 1 ms intervals could increase auditory nerve activities.

### Forward-Electrical Stimulation Interference in Intracochlear Electrocochleography of Acoustic Stimulation With Different Intensity

**FIGURE 5:**
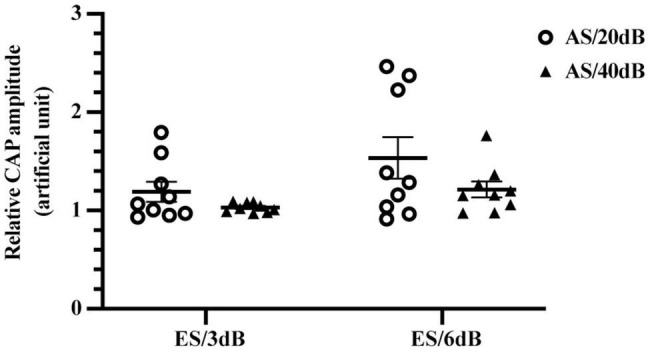
Forward-ES interference in iECochG of AS with different intensities. There was no significant difference between relative CAP amplitudes of 20 and 40 dB 4 kHz AS with 3 or 6 dB 1 ms-forward ES, *F*(1,16) = 3.074, *P* = 0.0987. Meanwhile, 6 dB forward ES produced higher relative CAP amplitudes than 3 dB ES in iECochG of both AS/20 dB and AS/40 dB, *F*(1,16) = 6.675, *P* = 0.0200. Each plot means an animal data and the horizontal line means the mean across animals. AS/20 dB and AS/40 dB, acoustic stimulation equivalent to 20 and 40 dB above iECochG thresholds, respectively. Treated with two-way RM ANOVA.

To further explore the impact of AS intensity on the forward-ES induced interference, we compared relative CAP amplitudes of 20 and 40 dB 4 kHz AS with 1 ms-forward 3 or 6 dB ES. As shown in Figure 5, there was no significant difference between relative CAP amplitudes of 20 and 40 dB AS with 3 or 6 dB ES, *F*(1,16) = 3.074, *P* = 0.0987. Meanwhile, 6 dB forward ES produced higher relative CAP amplitudes than 3 dB ES in iECochG of both 20 and 40 dB AS, *F*(1,16) = 6.675, *P* = 0.0200. These results indicated that 3 or 6 dB forward ES produced comparable elevations of CAP amplitudes in iECochG of 20 and 40 dB AS.

### Impacts of Electrical Stimulation Stimulating Electrode and Acoustic Stimulation Frequency on Forward Electrical Stimulation Interference in Intracochlear Electrocochleography of Acoustic Stimulation

**FIGURE 6:**
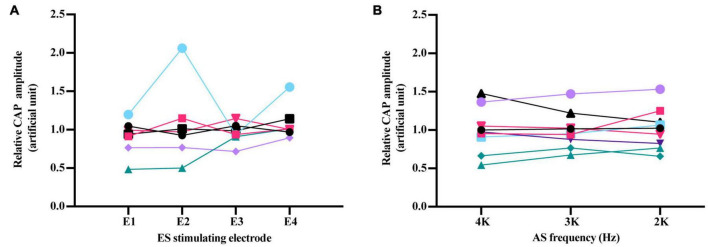
Impact of ES stimulating electrode and AS frequency on forward ES interference in iECochG of AS. **(A)** Relative CAP amplitudes of 4 kHz/20 dB AS were comparable when 1 ms-forward 3 dB ES was delivered by different electrodes, *F*(3,15) = 1.619, *P* = 0.2268. **(B)** With 1 ms-forward 3 dB ES, there was no significant difference among relative CAP amplitudes of AS at 4, 3, and 2 kHz, *F*(2,16) = 0.1413, *P* = 0.8693. Electrodes were marked E1–E4 from the apex to the base of the cochlea. Each line represented one experimental guinea pig. Repeated measure using one-way ANOVA. Each line with varied color and symbol represents one individual animal.

To investigate the impact of the spatial relationship between ES and AS on forward ES interference in AS responses, different electrodes (E1–E4) were used to deliver 1 ms-forward 3 dB ES combined 20 dB/4 kHz AS compared with corresponding iECochG (Figure 6A). Relative CAP amplitudes of these stimulating electrodes were all comparable [*F*(3,15) = 1.619, *P* = 0.2268]. Forward ES-induced interference in iECochG of 20 dB AS with different frequencies was also investigated (Figure 6B). With 1 ms-forward 3 dB ES, relative CAP amplitudes of 4, 3, and 2 kHz 20 dB AS were all comparable [*F*(2,16) = 0.1413, *P* = 0.8693]. These results suggest that the forward ES-induced interference in iECochG was not affected by the change of stimulating electrodes and acoustic frequencies in the current study.

## Discussion

Herein, we set up an EAS interaction detecting system by a combination of AEP and ECAP detecting devices and realized recording of iECochG and detecting of EAS interaction in the guinea pig cochlea. Using this system, for the first time, we observed that forward ES in the base of the cochlea could induce an elevation of CAP amplitudes of AS in the apical region. This effect was correlated with ES-AS interval and their intensities, but a not spatial relationship.

With the development of EAS application, understanding the EAS interaction becomes more important for a fitting strategy and an improvement of auditory performance. The EAS interaction detecting system established in this study could deliver combined EAS with adjustable intensity and interval. The delivered ES was single pulse paired to AS of single pure tone, while ES with pulse train coupled to AS was used in previous human studies (Koka and Litvak, 2017; Krüger et al., 2020a,b) and animal studies (Stronks et al., 2010, 2011, 2013). This study used iECochG to detect forward-ES induced interference in AS in animal studies for the first time. Previous studies have used extracochlear ECochG to assess the influence of extracochlear or intracochlear ES on AS (Stronks et al., 2011, 2013). Unlike certain intensities used in these studies, this study used the ES intensities based on corresponding ECAP thresholds to avoid unpredictable excitation of the cochlea and auditory nerve. The EAS interaction detecting system was also able to adjust the sequence of ES and AS and record ECAP to assess the influence of AS on ES. These capabilities made the system a proper objective tool to assess the peripheral electrophysiological characteristics of EAS interaction. In addition, with a customized intracochlear electrode array, the system could be used to investigate EAS interaction in animal models with specific pathological changes in the cochlea or auditory nerve.

Although the spatial relationship between the stimulating regions of 2, 3, and 4 kHz AS and recording electrode E1 location in the cochlea were different, this study did not find a significant difference among their CAP amplitudes. The intensities of the three ASs were 20 dB above their corresponding iECochG thresholds which were also determined by E1 recording. Even the same physical intensity of AS with different frequencies could produce different detected iECochG. Therefore, actual responses induced by the 2, 3, and 4 kHz 20 dB AS might be different. Another reason might be that CAP of the three ASs is mainly derived from the auditory nerve which has a constant spatial relationship with recording electrode E1. On the contrary, CAP amplitudes varied among non-adjacent recording electrodes, which might attribute to different spatial relationships of recording electrodes with AS stimulating region, auditory nerve, or both.

This study assessed the changes in CAP amplitudes to investigate the interaction between forward ES and afterward AS. Instead, previous human studies have used the changes in behavioral thresholds, CM/DIF, and ANN/SUM amplitudes to investigate EAS interaction (Koka and Litvak, 2017; Imsiecke et al., 2020a; Krüger et al., 2020a,b). Those studies suggest that CM/DIF amplitudes could decrease when AS was simultaneously presented with ES by pulse train. Meanwhile, ANN/SUM amplitudes measured in those studies showed no statistically significant attenuation with simultaneous ES (Koka and Litvak, 2017; Krüger et al., 2020b). ANN is phase-locked activity evoked by low-frequency AS (Kim, 2020). Therefore, at medium-high frequencies acoustic tests, ANN performs poorly. The existing studies focused on patients who have limited low-frequency residual hearing. Importantly, the results of studies on ANN are not significant enough and reliable. Interestingly, in support of our results, the mean amplitude attenuation of ANN/SUM was negative, representing an elevation of ANN/SUM amplitude (Krüger et al., 2020b). This study shows that the elevation of CAP amplitudes increased when ES intensities increased from 3 to 6 dB. Meanwhile, the elevation of CAP amplitudes in iECochG of 20 and 40 dB AS was comparable when either intensity of forward ES was applied. These enhancement effects of CAP amplitudes induced by forward ES might be attributed to accumulated activities of different auditory nerve fibers induced by ES and AS. Another possible reason might be ES-suppression of the spontaneous activity in those fibers that are afterward excited by AS.

In contrast to our results, previous animal studies using traditional ECochG suggest that the intracochlear ES at the basal turn of the cochlea could induce the suppression of CAP amplitudes at low frequencies (Stronks et al., 2011, 2013). However, the ES intensities used in those studies were usually 800 μA, while the intensities used in this study were determined based on the corresponding ECAP thresholds and had a mean of 349.769 μA for 3 dB ES. ES with much higher intensity than ECAP thresholds was supposed to have a broader exciting region of the cochlea which might overlap the stimulating region of AS. The dispersion effect of electrical current and complex conduction medium makes it hard to localize ES in the cochlea. Thus, hair cells in low-frequency areas may also be affected by high-intensity ES before AS. Given that ES levels used in CI recipients were also around ECAP thresholds, they were likely to produce a similar elevation of CAP amplitudes of AS stimulating many apical regions. In addition, the AS intensities mostly used in those studies were 80 dB SPL which were much higher than 24 dB SPL as a mean for 20 dB/4 kHz AS, as well as 64 dB SPL as a mean for 20 dB/4 kHz AS used in this study. AS with many high intensities might produce saturated CAPs whose amplitudes had no space to be elevated by ES. The elevation of CAP amplitudes in iECochG of 20 and 40 dB was comparable when either 3 or 6 dB ES was combined with the ES. It indicated that CAP amplitudes derived from these EAS were not in a state of saturation.

Several studies on electrical and frequency difference (EAFD) made a great contribution to the intensity of interaction (Krüger et al., 2017; Imsiecke et al., 2018, 2020b). They found that the bigger overlapping area of ES and AS was, the more significant the interaction was. Unlike their experiment, our electrode applied on the cochlea of a guinea pig is shorter. The ES area in our study did not exceed the basal part of the cochlea. Therefore, the distance from ES electrode to AS frequency area may also result in no significant CAP amplitude change.

The EAS interaction system still had some limitations. Temporal accuracy in this study is 1 ms, limited by the hardware conditions. As the duration of ARP and RRP can be divided into microseconds, further study of intervals should employ a more exquisite system that can stimulate and sample more accurately. Moreover, a single sample period in this system is too short for a complete sampling of ECochG. This study used a splicing strategy to obtain a complete waveform of iECochG. An improvement of hardware for a long enough sampling period would increase accuracy and save time in iECochG recording. The system speed of ES traveling in tissue is close to the light, while the speed of AS is more complex. In a different medium, AS travels at a different speed. From insert earphones, AS arouse lymph and basement membrane vibration in the cochlea through air conduction and bone conduction. The path has an inherent delay affecting the accuracy of an actual interval between ES and AS (Chung et al., 2016). This study is a short-term animal experiment. Obviously, further electrophysiological studies in human CI recipients are needed to extend our knowledge on the interaction of non-simultaneous EAS.

## Conclusion

This study successfully established a system allowing ES and AS output synchronously with adjustable intensity and interval, which can investigate the interaction between AS in the apical region and ES in the basal region of the cochlea. Our results suggest that forward ES at the basal region of the cochlea could induce the elevations of CAP amplitudes in iECochG of AS at the apical region. The elevations of CAP amplitudes were supposed to be derived from accumulated activities of different auditory nerve fibers induced by ES and AS in different regions of the cochlea.

## Data Availability Statement

The raw data supporting the conclusions of this article will be made available by the authors, without undue reservation.

## Ethics Statement

The animal study was reviewed and approved by the Ethics Committee of Eye & ENT Hospital of Fudan University.

## Author Contributions

SM: data curation, formal analysis, investigation, methodology, visualization, and writing – original draft. TL, MC, JM, and XH: investigation, methodology, and writing – review. SL: conceptualization, formal analysis, funding acquisition, methodology, project administration, resources, supervision, visualization, and writing. All authors approval of final version of submitted manuscript.

## Conflict of Interest

The authors declare that the research was conducted in the absence of any commercial or financial relationships that could be construed as a potential conflict of interest.

## Publisher’s Note

All claims expressed in this article are solely those of the authors and do not necessarily represent those of their affiliated organizations, or those of the publisher, the editors and the reviewers. Any product that may be evaluated in this article, or claim that may be made by its manufacturer, is not guaranteed or endorsed by the publisher.
